# *Wolbachia* strain *w*AlbA blocks Zika virus transmission in *Aedes aegypti*

**DOI:** 10.1111/mve.12384

**Published:** 2019-05-23

**Authors:** T. Chouin‐Carneiro, T. H. Ant, C. Herd, F. Louis, A. B. Failloux, S. P. Sinkins

**Affiliations:** ^1^ Department of Virology, Arboviruses and Insect Vectors Institut Pasteur Paris France; ^2^ MRC‐University of Glasgow Centre for Virus Research University of Glasgow Glasgow U.K.; ^3^ Biomedical and Life Sciences Lancaster University Lancaster U.K.

**Keywords:** *Aedes aegypti*, *Aedes albopictus*, dengue virus arbovirus, pathogen blocking, *Wolbachia*, Zika virus

## Abstract

Transinfections of the maternally transmitted endosymbiont *Wolbachia pipientis* can reduce RNA virus replication and prevent transmission by *Aedes aegypti*, and also have the capacity to invade wild‐type populations, potentially reaching and maintaining high infection frequencies. Levels of virus transmission blocking are positively correlated with *Wolbachia* intracellular density. Despite reaching high densities in *Ae. aegypti*, transinfections of *w*AlbA, a strain native to *Aedes albopictus*, showed no blocking of Semliki Forest Virus in previous intrathoracic injection challenges. To further characterize *w*AlbA blocking in *Ae. aegypti*, adult females were intrathoracically challenged with Zika (ZIKV) and dengue viruses, and then fed a ZIKV‐containing bloodmeal. No blocking was observed with either virus when challenged by intrathoracic injection. However, when ZIKV was delivered orally, *w*AlbA‐infected females showed a significant reduction in viral replication and dissemination compared with uninfected controls, as well as a complete absence of virus in saliva. Although other *Wolbachia* strains have been shown to cause more robust viral blocking in *Ae. aegypti*, these findings demonstrate that, in principle, *w*AlbA could be used to reduce virus transmission in this species. Moreover, the results highlight the potential for underestimation of the strength of virus‐blocking when based on intrathoracic injection compared with more natural oral challenges.

## Graphical abstract

- A transinfection of the *w*AlbA *Wolbachia* strain in *Aedes aegypti* blocks Zika virus transmission in saliva when virus is delivered orally via an infectious bloodmeal.
- Significant Zika blocking by *w*AlbA was not observed when virus was delivered via intrathoracic injection, suggesting that the midgut barrier plays an important role in blocking.
- Intrathoracic challenges can misrepresent virus blocking strength and may underestimate the true blocking potential of a strain.

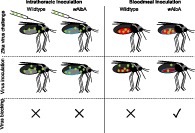

*Wolbachia* are maternally‐transmitted alphaproteobacteria widespread among the phylum Arthropoda. These endosymbionts are obligately intracellular, comprising a large number of distinct strains distributed among a wide diversity of host species. *Wolbachia* strains are currently classified into a set of 16 phylogenetically distinct supergroups (A–Q) (Glowska *et al*., 2015; Gerth, 2016), with supergroups A and B containing strains capable of causing host reproductive parasitism (Casiraghi *et al*., 2005; Bordenstein *et al*., 2009; Zu Dohna *et al*., 2018).

*Wolbachia* are currently being deployed in the field as a vector control intervention. Certain *Wolbachia* strains cause a strong reduction in vector competence for RNA viruses, particularly when novel *Wolbachia*–host combinations are generated (Moreira *et al*., 2009; Bian *et al*., 2010; Kambris *et al*., 2010; Walker *et al*., 2011; Blagrove *et al*., 2012; van den Hurk *et al*., 2012; Joubert *et al*., 2016; Fraser *et al*., 2017; Ant *et al*., 2018). In the primary DENV vector *Aedes aegypti*, for example, *Wolbachia* transinfected lines have shown strong transmission blocking for the major arboviruses, including dengue (DENV) (Moreira *et al*., 2009; Walker *et al*., 2011; Frentiu *et al*., 2014; Ant *et al*., 2018), chikungunya (Moreira *et al*., 2009; van den Hurk *et al*., 2012), Zika (ZIKV) (Aliota *et al*., 2016; Dutra *et al*., 2016; Ant *et al*., 2018) and yellow fever (van den Hurk *et al*., 2012)*. Wolbachia* density is generally higher, and tissue distribution broader, in novel transinfections compared with naturally occurring host–*Wolbachia* associations, and this is considered to the enhance the transmission blocking phenotype (Lu *et al*., 2012; Osborne *et al*., 2012; Chrostek *et al*., 2013; Martinez *et al*., 2014).

The host reproductive manipulations generated by some *Wolbachia* strains facilitates their population invasion and the maintenance of high infection frequencies. Cytoplasmic incompatibility (CI) is a sperm modification that results in sterility unless a compensatory *Wolbachia* rescue factor is present in the embryo. The coupling of CI rescue with maternal transmission generates a relative reproductive advantage for *Wolbachia‐*infected females, with frequency thresholds for population invasion largely determined by the balance between the fitness benefits of CI and any negative effects on life history (Turelli & Hoffmann, 1999; Jansen *et al*., 2008; Turelli, 2010; Hancock *et al*., 2011; Hancock *et al*., 2016).

The invasive arbovirus vector *Aedes albopictus* is naturally superinfected with the *w*AlbA (supergroup A) and *w*AlbB (supergroup B) *Wolbachia* strains, where *w*AlbA tends to exist at a low intracellular density relative to *w*AlbB (Dutton & Sinkins, 2004) and is hypothesized to have a longer evolutionary association with *Ae. albopictus* (Sinkins *et al*., 1995). A transinfection of both strains generated in *Ae. aegypti* revealed a reversal of the relative strain densities in this novel host, with *w*AlbA displaying broad tissue distribution and higher densities in somatic tissues compared with *w*AlbB, suggesting that the line would show strong virus inhibition (Ant *et al*., 2018). However, when wAlbA‐carrying females were challenged with Semliki Forest Virus (SFV) via thoracic injection, no reduction in viral genome copies was detected compared with *Wolbachia*‐free controls (Ant *et al*., 2018). In the present study, further characterization of the viral blocking capacity of *w*AlbA in *Ae. aegypti* is provided via challenge by intrathoracic injection with ZIKV and DENV viruses and oral feeding of ZIKV.

For the intrathoracic challenges, 30 5‐day old female mosquitoes from the *w*AlbA, *w*Au and wild‐type lines were injected with either DENV or ZIKV in the thorax using a pulled glass capillary and a Nanoject II (Drummond Scientific, Broomall, PA, U.S.A.) hand‐held microinjector. Injected mosquitoes were immediately transferred to an incubator set to 27 °C for recovery. DENV injected females were left for 10 days prior to RNA extraction and virus quantification by a quantitative reverse transcriptase‐polymerase chain reaction. ZIKV injected females were left for 7 days. DENV was serotype 2, New Guinea C strain, obtained from Public Health England culture collections. The concentration of injected DENV virus was 2.5 × 10^8^ FFU/mL. ZIKV was strain MP1751, obtained from Public Health England culture collections. The concentration of injected ZIKV virus was 4.8 × 10^8^ FFU/mL. The primers used to measure DENV genome copies were DENV‐NS5‐F: 5′‐ACAAGTCGAACAACCTGGTCCAT‐3′ and DENV‐NS5‐R: 5′‐GCCGCACCATTGGTCTTCT‐3. The primers used to measure ZIKV genome copies were ZIKV‐835: 5′‐TTGGTCATGATACTGCTGATTG‐3′ and ZIKV‐911c: 5′‐CCTTCCACAAAGTCCCTATTGC‐3′.

For the oral infections, 7‐day‐old *w*AlbA and wild‐type females were fed an infectious blood‐meal containing 1.4 mL of washed rabbit erythrocytes and 700 μL of viral suspension supplemented with ATP at a final concentration of 5 mm. The day before the infectious blood‐meal, batches of 65 females were isolated in feeding boxes and starved for 24 h. Mosquitoes were then exposed to the ZIKV NC‐2014‐5132 strain containing a final viral titre of 10^7^ TCID_50_/mL.

For the infection, dissemination and transmission analysis, population batches of 30 *w*AlbA and 30 wild‐type mosquitoes were analysed at days 4, 7, 11, 14 and 21 post infection. To estimate infection, dissemination and transmission, mosquito bodies (thorax and abdomen), heads and saliva were analysed, respectively. To assess the transmission rate and transmission efficiency, mosquito saliva was collected from individual mosquitoes. Infection rate was determined by the proportion of mosquitoes with infected body (abdomen and thorax) among tested mosquitoes. The disseminated infection rate corresponds to the proportion of mosquitoes with infected heads among the previously detected infected mosquitoes (i.e. abdomen/thorax positive). The transmission rate refers to the proportion of mosquitoes with infectious saliva among mosquitoes with disseminated infection. Virus was titrated by plaque assay.

The capacity of the *w*AlbA strain to inhibit ZIKV and DENV in *Ae. aegypti* was assessed via virus intrathoracic injection and was compared with the blocking capacity of *w*Au, a strain with comparable intracellular densities but that had previously shown strong viral inhibition (Ant *et al*., 2018). Consistent with the findings for SFV, genome copies in *w*AlbA females did not differ significantly from *Wolbachia‐*uninfected wild‐type mosquitoes (for DENV *P* = 0.636, Wilcoxon rank sum; for ZIKV, *P* = 0.057, Wilcoxon rank sum). The *w*Au‐carrying line showed significantly reduced levels of both viruses (for DENV, *P* < 0.0001, Wilcoxon rank sum; for ZIKV, *P* < 0.00001, Wilcoxon rank sum), with 16 out of 24 (66.7%) ZIKV injected females containing no detectable virus compared with 100% virus positivity in wild‐type and *w*AlbA‐infected mosquitoes (Fig. 1).

**Figure 1:**
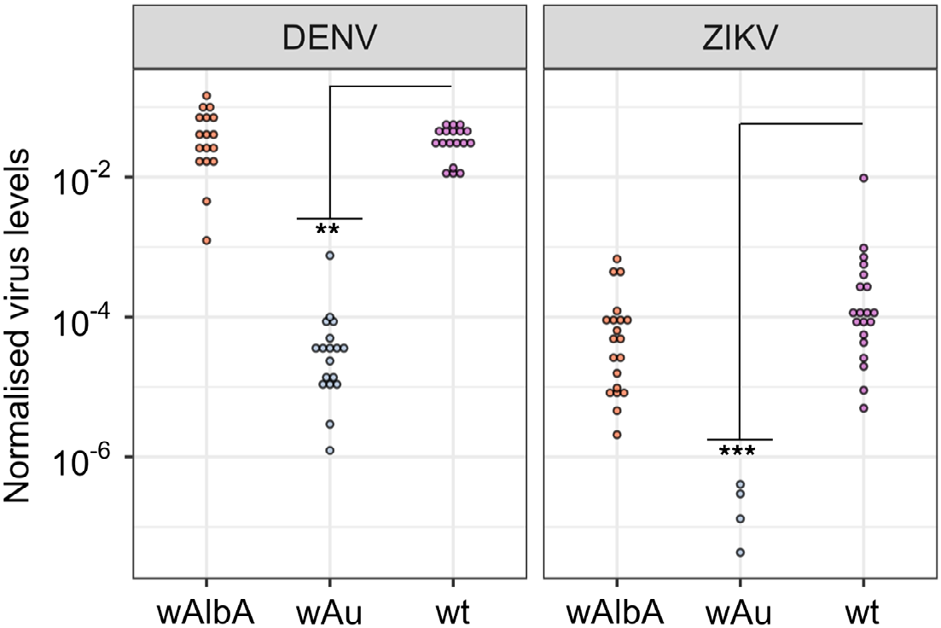
Dengue (DENV) and Zika (ZIKV) viral genome copies per host cell after thoracic injection into *Wolbachia*‐infected lines and wild‐type *Aedes aegypti*. Females were left for 10 days prior to total RNA extraction and virus quantification by a quantitative polymerase chain reaction. The amount of virus for each female was normalized to the *RpS17* house‐keeping gene. Statistical analysis was performed using a Wilcoxon rank sum test with *P* < 0.05 considered statistically significant. [Colour figure can be viewed at http://wileyonlinelibrary.com].

To assess the blocking potential of the *w*AlbA strain when orally challenged, *w*AlbA‐carrying and wild‐type females were fed a blood meal containing ZIKV. Rates of ZIKV infection in whole females, viral dissemination to head, legs or wings, and the presence of infectious virus in saliva were assessed by plaque assay over a course of 21 days post infectious blood meal. Significant and consistent reductions were observed in the rates of whole female infectivity and virus dissemination in the *w*AlbA line compared with wild‐type, although these reductions were fairly modest, consistent with the low levels of blocking observed in the intrathoracic infections. However, the *w*AlbA infection in *Ae. aegypti* caused complete blocking of virus transmission (i.e. an absence of detectable virus in saliva), whereas wild‐type females were capable of transmitting infectious virus after 14 days post infectious blood meal (Fig. 2). Differences in transmission at day 14 were found to be significantly lower in *w*AlbA infected females (*P* < 0.05, Fisher's exact test).

**Figure 2:**
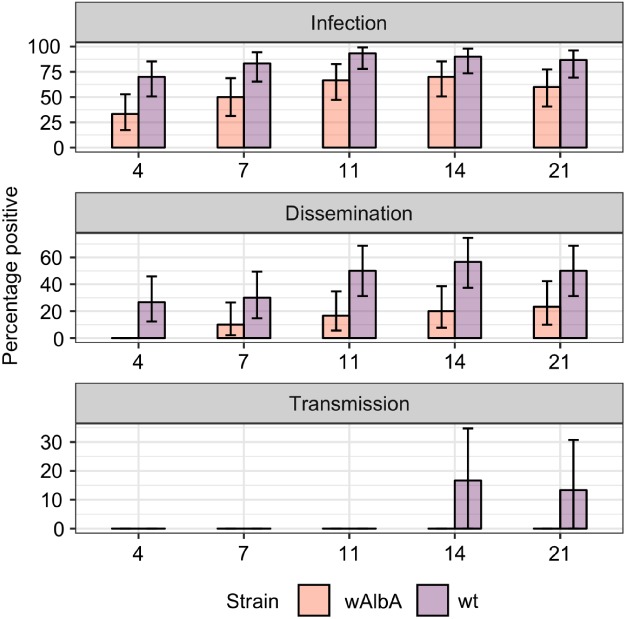
Percentage of females testing positive for Zika (ZIKV) infection, ZIKV dissemination to either the head, legs or wings, or ZIKV positivity in saliva measured by plaque assay. Each bar shows the percentage rates from 30 infected females of each strain with error bars showing the binomial 95% confidence intervals. Statistical testing was performed using a one‐tailed Fisher's exact test with *P* < 0.05 considered statistically significant. [Colour figure can be viewed at http://wileyonlinelibrary.com].

The difference in levels of virus inhibition between the intrathoracic and oral challenges highlights the biologically crucial role of the midgut and salivary gland barriers in *Wolbachia*‐mediated virus transmission blocking. Although present at lower densities than in salivary gland or ovary tissues, *w*AlbA is found in the cells of the *Ae. aegypti* midgut epithelium (Ant *et al*., 2018). The reduced dissemination of Zika virus to the legs or wings in the *w*AlbA line suggests that, even at a relatively low level, *Wolbachia* can limit the ability of ZIKV to cross the midgut barrier and escape into the haemolymph. Intrathoracic inoculation artificially bypasses the midgut cells, introducing high viral titres directly into the haemocoel. The data reported in the present study therefore highlight the importance of conducting oral challenges when assessing the potential of different *Wolbachia* strains as arbovirus control agents. Although technically easier to conduct, intrathoracic virus challenges alone can produce misleading results with respect to transmission‐blocking potential. Moreover, these findings illustrate the greater resolution achieved when virus blocking is characterized in terms of head dissemination, as well as virus titres in saliva, compared with whole‐body quantification. *w*AlbA can now be added to the small but growing list of *Wolbachia* strains that have been demonstrated to block transmission of ZIKV in *Ae. aegypti* and also have potential as tools for use in arbovirus control. Given the variability in blocking capacity and considering that comparative fitness effects can vary under different environmental conditions such as ambient temperature (Ulrich *et al*., 2016; Ross *et al*., 2017; Ant *et al*., 2018), it is important to create a range of *Ae. aegypti* transinfections with as many strains and therefore phenotypes as possible.
